# Potent HIV-1-Specific CD8 T Cell Responses Induced in Mice after Priming with a Multiepitopic DNA-TMEP and Boosting with the HIV Vaccine MVA-B

**DOI:** 10.3390/v10080424

**Published:** 2018-08-13

**Authors:** Beatriz Perdiguero, Suresh C. Raman, Cristina Sánchez-Corzo, Carlos Oscar S. Sorzano, José Ramón Valverde, Mariano Esteban, Carmen Elena Gómez

**Affiliations:** 1 Department of Molecular and Cellular Biology, Centro Nacional de Biotecnología, Consejo Superior de Investigaciones Científicas (CNB-CSIC), Campus de Cantoblanco, 28049 Madrid, Spain; perdigue@cnb.csic.es (B.P.); schithathur@cnb.csic.es (S.C.R.); cscorzo@cnb.csic.es (C.S.-C.); 2 Biocomputing Unit, Centro Nacional de Biotecnología, Consejo Superior de Investigaciones Científicas (CNB-CSIC), Campus de Cantoblanco, 28049 Madrid, Spain; coss@cnb.csic.es; 3 Scientific Computing Service, Centro Nacional de Biotecnología, Consejo Superior de Investigaciones Científicas (CNB-CSIC), Campus de Cantoblanco, 28049 Madrid, Spain; jrvalverde@cnb.csic.es

**Keywords:** multiepitopic vaccine, MVA, HIV-1, immunogenicity

## Abstract

An effective vaccine against Human Immunodeficiency Virus (HIV) still remains the best solution to provide a sustainable control and/or eradication of the virus. We have previously generated the HIV-1 vaccine modified vaccinia virus Ankara (MVA)-B, which exhibited good immunogenicity profile in phase I prophylactic and therapeutic clinical trials, but was unable to prevent viral rebound after antiretroviral (ART) removal. To potentiate the immunogenicity of MVA-B, here we described the design and immune responses elicited in mice by a new T cell multi-epitopic B (TMEP-B) immunogen, vectored by DNA, when administered in homologous or heterologous prime/boost regimens in combination with MVA-B. The TMEP-B protein contained conserved regions from Gag, Pol, and Nef proteins including multiple CD4 and CD8 T cell epitopes functionally associated with HIV control. Heterologous DNA-TMEP/MVA-B regimen induced higher HIV-1-specific CD8 T cell responses with broader epitope recognition and higher polyfunctional profile than the homologous DNA-TMEP/DNA-TMEP or the heterologous DNA-GPN/MVA-B combinations. Moreover, higher HIV-1-specific CD4 and Tfh immune responses were also detected using this regimen. After MVA-B boost, the magnitude of the anti-VACV CD8 T cell response was significantly compromised in DNA-TMEP-primed animals. Our results revealed the immunological potential of DNA-TMEP prime/MVA-B boost regimen and supported the application of these combined vectors in HIV-1 prevention and/or therapy.

## 1. Introduction

The access to antiretroviral therapy (ART) among 53% of all people living with HIV has had a significant impact on disease progression and transmission. Deaths by Acquired Immune Deficiency Syndrome (AIDS)-related causes have dropped from a peak of 1.9 million in 2005 to 1.0 million in 2016. However, the global burden of HIV is still alarming. At the end of 2016, over 36 million people worldwide were living with HIV/AIDS (http://www.unaids.org/en/resources/documents/2017/2017_data_book). These statistics highlight the need for an effective vaccine that could provide a sustainable solution for the control and/or eradication of the virus.

The overall immune correlates of protection from HIV are not well established, but there is a consensus that a successful vaccine needs to stimulate both humoral and cellular immune responses, covering the global HIV diversity. Despite all the efforts done in the search for an ideal and versatile vaccine, none of the candidates have proven by themselves to efficiently potentiate both immunological arms. For this reason, present studies have shifted towards the search for individual immunogens that can independently induce broadly neutralizing antibodies or elicit an effective cellular T cell response, and combining them in heterologous immunization regimens to trigger virus neutralization and pathogen-infected cell elimination.

We have previously shown, in a phase-I double-blinded placebo-controlled trial (RISVAC02), that homologous prime-boost vaccination of healthy volunteers with three doses of modified vaccinia virus Ankara (MVA)-B vector expressing the HIV-1 Env, Gag, Pol, and Nef antigens from clade B was safe, well tolerated, and elicited moderate and durable (up to one year) HIV-1-specific T cell and antibody responses mainly directed against the Env antigen [1,2]. After four years, 20% of vaccinees maintained low HIV-1-specific T cell responses, suggesting that three doses of MVA-B did not induced long-term T cell memory response against HIV infection. However, a late MVA-B boost significantly increased the binding and neutralizing antibody responses [3]. In chronic HIV-1-infected individuals, vaccination with MVA-B, given alone or in combination with disulfiram, did not have a major impact on the latent reservoir or the rebound of plasma viral load after cART interruption [4,5].

To avoid the immunodominance of Env and to potentiate the MVA-B-induced responses, we designed a T cell multiepitopic-based immunogen including those regions of Gag, Pol, and Nef proteins that have been functionally associated with HIV control instead of the full-length antigens. An epitope-based vaccine is described to have many advantages over other types of vaccines including safety, immunogenicity, and population coverage. It allows the inclusion of those antigenic regions relevant for protection that can bind to a maximum number of Human Leukocyte Antigen (HLA) molecules to cover the entire target population’s HLA diversity and the exclusion of sequences related with virulence [6].

Different strategies for antigen design have been implemented thus far to elicit protective and durable T cell immune responses tackling HIV-1 diversity. These strategies mostly include the use of ‘Mosaic’ immunogens based on intact proteins that optimize the coverage of T cell epitopes, the use of beneficial regions associated with HIV control defined by human immune reactivity data, and/or the use of highly conserved regions of the HIV-1 consensus proteome [7,8,9,10,11,12]. The immunogens based on highly conserved elements of HIV-1 were developed under the premise that mutations at conserved regions would have a high fitness cost, potentially driving the virus to less fit forms that would be better controlled by the immune system. Furthermore, they modify the immunodominance hierarchies observed in HIV infection and in vaccines encoding full-length HIV proteins by inducing T cell-mediated responses to subdominant conserved epitopes [7,9,13]. The last generation of T cell immunogens combines all or most of the above strategies and has been assayed in preclinical and clinical trials [11,12,14,15].

Here, we applied the most advanced antigen design strategies described above to generate a new T cell HIV-1 immunogen, termed T cell multi-epitopic B (TMEP-B), containing eight HIV-1 segments, mostly derived from conserved regions in Gag, Pol, and Nef proteins, that included multiple beneficial CD4 and CD8 T cell epitopes restricted by a wide range of HLA class I and II molecules that have been functionally associated with low viral load and HIV control. The immunogenicity of TMEP-B protein, vectored by DNA, was evaluated in mice in homologous or heterologous combination with the MVA-B vaccine that simultaneously expresses the monomeric gp120 and the full-length HIV-1 Gag–Pol–Nef (GPN) fusion protein.

## 2. Materials and Methods

### 2.1. Synthetic TMEP-B Gene

The TMEP-B protein sequence was translated into an RNA/codon optimized nucleotide sequence avoiding RNA processing, inhibitory, and instability elements, as well as predicted splice sites. The synthetic TMEP-B gene (1868 bp) was assembled from synthetic oligonucleotides and/or PCR products and was preceded by a consensus Kozak sequence at six nucleotides to maximize protein expression. The fragment was inserted into the transfer vector pCyA-20 [16] by Invitrogen (Carlsbad, CA, USA), generating the pCyA-20-TMEP-B plasmid.

### 2.2. Frequencies of TMEP-B Epitopes in the General HIV-1-Infected Population and in Long Term Non-Progressors (LTNPs)

The information for all known HIV-1 *gag*, *pol*, and *nef* genes, their translated products and all the CTL/CD8^+^ and T helper CD4^+^ epitopes and variants were retrieved from Los Alamos HIV databases (https://www.hiv.lanl.gov) [17]. All reported sequences were obtained from NCBI (REF: NCBI Resource Coordinators (2016). Database resources of the National Centre for Biotechnology Information (NCBI). Nucleic acids research, 44 (Database issue), D7). The databases were accessed on 4 March 2018.

Matches for CTL/CD8^+^ and T helper CD4^+^ T cell epitopes were identified in sequences from the general HIV-1-infected population and in LTNP patients using Blast [18]. The relative frequencies of each epitope sequence in both populations were computed as the absolute frequency divided by the number of individuals considered, and were then compared.

### 2.3. Cells and Viruses

The highly transfectable 293T cell line, derived from human epithelial embryonic kidney 293 cells containing the SV40 T-antigen, was grown in Dulbecco’s modified Eagle’s medium (DMEM) supplemented with 100 μg/mL streptomycin, 100 U/mL penicillin (both from Invitrogen), 2 mM l-glutamine (Merck, Kenilworth, NJ, USA) and 10% fetal calf serum (FCS; Sigma-Aldrich, St. Louis, MO, USA) and maintained in a humidified air 5% CO_2_ atmosphere at 37 °C. The poxvirus strains used in this study included the vaccinia virus (VACV) Western Reserve strain (WR), the previously described inducible recombinant VACV that expresses the T7 RNA polymerase (VT7) [19], the attenuated wild-type modified vaccinia virus Ankara (MVA-WT) obtained from the Ankara strain after 586 serial passages in chicken embryo fibroblast (CEF) cells (kindly provided by G. Sutter) and the recombinant MVA-B virus that simultaneously expresses the monomeric HIV-1_BX08_ gp120 protein as a cell-released product and the artificial budding defective 1326 aa read-through HIV-1_IIIB_ Gag–Pol–Nef (GPN) fusion protein as an intracellular product [20]. After infection, complete DMEM supplemented with 2% FCS was added to cultured cells.

### 2.4. DNA Vectors

**Figure 1:**
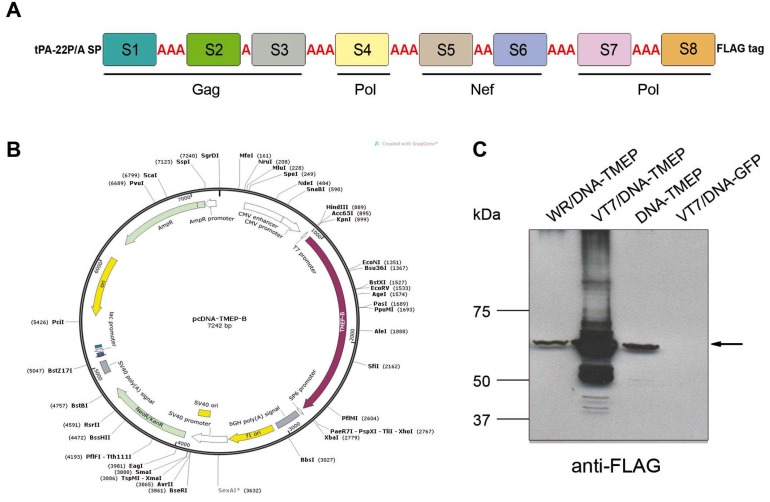
T cell multiepitopic B (TMEP-B) design, construction of pcDNA-TMEP-B plasmid, and TMEP-B expression analysis by Western blot. (**A**) Scheme of TMEP-B protein. (**B**) Map of the plasmid pcDNA-TMEP-B. (**C**) Expression of TMEP-B construct by Western blot. The 293T cells were mock-infected or infected with 5 pfu/cell of Western Reserve (WR) or vaccinia virus (VACV) that expresses the T7 RNA polymerase (VT7) viruses, and transfected 1 h later with 5 μg of pcDNA-TMEP-B or pMax-GFP. At 6 h post-infection, cells were harvested and lysed in Laemmli buffer with mercapoethanol and cell extracts were fractionated by 8% SDS-PAGE and analyzed by Western blot using mouse monoclonal anti-FLAG M2 antibody to evaluate TMEP-B expression.

For the generation of the pcDNA-TMEP-B vector, the synthetic TMEP-B gene was excised from plasmid pCyA-20-TMEP-B with the restriction endonucleases KpnI and XhoI and inserted into the pcDNA3.0 vector (previously digested with KpnI+XhoI; Invitrogen) between the human cytomegalovirus (CMV) promoter and bovine growth hormone (BGH) polyadenylation signal (Figure 1B).

The DNA construct expressing the HIV-1_IIIB_ GPN fusion protein (pcDNA-_IIIB_GPN) has been previously reported [20]. Plasmids pcDNA-TMEP-B (DNA-TMEP) and pcDNA-_IIIB_GPN (DNA-GPN) were purified using the EndoFree Plasmid Mega kit (Qiagen, Hilden, Germany) and diluted for injection in endotoxin-free phosphate-buffered saline (PBS).

### 2.5. Transfection Assay and Expression of TMEP-B Protein by Western Blot Analysis

To determine the correct expression of TMEP-B protein from DNA-TMEP vector, 293T cells (1 × 10^6^) were mock-infected or infected with 5 pfu/cell of WR or VT7 viruses and transfected 1 h later with 5 μg of DNA-TMEP or pMax-GFP (Lonza, Basel, Switzerland) using Lipofectamine 2000 (Invitrogen) according to manufacturer’s recommendations. At 6 h post-infection, cells were harvested, washed with PBS and lysed in Laemmli buffer with β-mercaptoethanol; cell extracts were fractionated by 8% SDS-PAGE and analyzed by Western blot using mouse monoclonal anti-FLAG M2 antibody (1:1000; Sigma-Aldrich) to evaluate TMEP-B expression. Anti-mouse-horseradish peroxidase (1:2000; SIGMA-ALDRICH) was used as conjugated secondary antibody. Immune complexes were observed by enhanced chemiluminescence system (ECL; GE Healthcare, Chicago, IL, USA).

### 2.6. Ethics Statement

Animal experimental protocols were approved by the Ethical Committee of Animal Experimentation of Centro Nacional de Biotecnología (CEEA-CNB, Madrid, Spain) according to International European Union (EU) Guidelines 2010/63/UE on protection of animals used for experimentation and other scientific purposes, Spanish National Royal Decree RD 1201/2005 and Spanish National Law 32/2007 on animal welfare, exploitation, transport, and sacrifice (permit number PROEX 331/14).

### 2.7. Mouse Immunization Schedule

BALB/c mice were purchased from Harlan. Groups of 6–8-week-old female mice (*n* = 4) were primed with 50 μg of DNA-GPN, DNA-TMEP, or sham pcDNA vector (DNA-φ) by bilateral intramuscular route (i.m.). Three weeks later, they were boosted by bilateral i.m. inoculation with either 50 μg of DNA (DNA-GPN, DNA-TMEP, or DNA-φ) or 1 × 10^7^ pfu of virus (MVA-WT or MVA-B). At 10 days after the last immunization, mice were sacrificed, and spleens and draining lymph nodes (DLNs) were processed for intracellular cytokine staining assay to analyze antigen-specific cellular immune responses, as well as anti-vector immunity. Sera were harvested and used to analyze humoral immune responses to p24 antigen by ELISA. Representative data are shown for two experiments.

### 2.8. Peptides

The HIV-1 clade B consensus peptide pools Env-1 (63 peptides), Env-2 (61 peptides), Gag-1 (55 peptides), Gag-2 (50 peptides), GPN-1 (53 peptides), GPN-2 (57 peptides), GPN-3 (56 peptides), and GPN-4 (55 peptides) were provided by the NIH AIDS Research and Reference Reagent Program (Bethesda, MD, USA). They spanned the HIV-1 Env, Gag, Pol, and Nef antigens from clade B included in the immunogens expressed by MVA-B as consecutive 15-mers overlapping by 11 amino acids. The pools that span the different fragments included in TMEP-B construct are indicated in Table 1. To analyze the HIV-1-specific cellular immune responses, we combined the different peptide pools as follows: Env pool (Env-1+Env-2), Gag-1 pool, Gag-2 pool, GPN-1 pool, GPN-2 pool, GPN-3 pool, and GPN-4 pool. The VACV E3_140-148_ peptide (VGPSNSPTF; CNB-CSIC Proteomics Service), previously described as an immunodominant epitope in BALB/c mice [21], was used to determine VACV-specific CD8 T cell responses.

### 2.9. Intracellular Cytokine Staining (ICS) Assay

The magnitude and phenotype of HIV-1- or VACV-specific T cell responses were analyzed by ICS. After an overnight rest, 2 × 10^6^ splenocytes or lymphocytes from DLNs (erythrocyte-depleted) were seeded on 96-well plates and stimulated for 6 h in supplemented RPMI 1640 medium with 10% FCS, 1 μL/mL Golgiplug (BD Biosciences, Franklin lakes, NJ, USA) and 5 μg/mL of HIV-1 clade B peptide pools or 10 μg/mL of VACV E3 peptide. After the stimulation period, cells were washed, stained for surface markers, permeabilized (Cytofix/Cytoperm kit; BD Biosciences), and stained intracellularly with appropriate fluorochromes. For the analysis of CD4 and CD8 T cell immune responses, fluorochrome-conjugated antibodies used were the following: CD3-PECF594, CD4-APCCy7, CD8-V500, CD107a-FITC, IL-2-APC, IFN-γ-PeCy7, and TNF-α-PE for functional analyses and CD127-PerCPCy5.5 and CD62L-Alexa 700 for phenotypic analyses. For the analysis of Tfh cell immune responses, fluorochrome-conjugated antibodies used were the following: CD4-Alexa 700, CD8-V500, CD154 (CD40L)-Biotin/Avidin-PE, IL-4-FITC, IFN-γ-PeCy7, and IL-21-APC for functional analyses and CXCR5-PECF594, PD1 (CD279)-APCefluor780, and CD44-PeCy5 (SPRD) for phenotypic analyses. All antibodies were from BD Biosciences. Dead cells were excluded using the violet LIVE/DEAD stain kit (Invitrogen). Cells were acquired in a GALLIOS flow cytometer (Beckman Coulter, Brea, CA, USA) and data analyses were performed using FlowJo software (Version 10.4.2; Tree Star, Ashland, OR, USA). The number of lymphocyte-gated events ranged between 10^5^ and 5 × 10^5^. After lymphocyte gating, Boolean combinations of single functional gates were created using FlowJo software to quantify the frequency of each response based on all possible combinations of cytokine expression or differentiation marker expression. For every specific functional combination, background responses obtained in the non-stimulated control samples (RPMI) were subtracted from those detected in stimulated samples, and the percentages of cells producing cytokines in the control groups were also subtracted from all groups to remove the non-specific responses.

### 2.10. Antibody Measurement by Enzyme-Linked Immunosorbent Assay (ELISA)

Antibody binding to Gag-p24 protein in serum was assessed by ELISA as previously described [20]. Sera from immunized mice were diluted 1:50 in duplicate and incubated with 2 μg/mL of recombinant p24 from HXB2 strain produced in *Pichia pastoris* (ARP678, HIV-1 p24 clade B; EU Programme EVA, NIBSC Centralised Facility for AIDS Reagents). Levels of binding anti-p24 IgG antibodies were defined as the optical density measured at 450 nm (OD_450_ value). The data of p24-specific humoral response comprises the values obtained in two different in vivo assays (8 mice per group in total).

### 2.11. Data Analysis and Statistics

For the statistical analysis of ICS data, we corrected measurements for the non-stimulated control samples (RPMI) and calculated confidence intervals and *p* values of hypothesis tests as previously described [22]. Only antigen response values significantly higher than the RPMI value are represented; background for the different cytokines in non-stimulated control samples were never >0.05%. Analysis and presentation of distributions in the polyfunctional response were performed by using SPICE version 5.1 software [23].

For statistical analysis of ELISA data, we performed a one-way analysis of variance (ANOVA) test followed by Tukey’s honest significant difference criterion.

## 3. Results

### 3.1. Design of the T Cell Multiepitopic Peptide TMEP-B Immunogen

During recent years, several synthetic epitope-based immunogens using regions of HIV-1 proteins functionally conserved across all M group viruses and/or associated with viral control have been successfully tested in preclinical and clinical studies [7,8,10,11,12,15]. To extend this approach, here we designed a new T cell multiepitopic peptide, termed TMEP-B, as an HIV-1 immunogen. The fragments included were selected based on several criteria: (i) inclusion of regions in the HIV-1 proteome functionally conserved across all clades that contain beneficial CD8^+^ T cell epitopes associated with low viral load with exclusion of immunodominant decoy epitopes that are irrelevant for HIV control; (ii) presence of relevant CD4^+^ T helper epitopes; and (iii) coverage of a wide range of the most common allelic variants of HLA class I and class II molecules.

In total, eight HIV-1 regions, ranging from 13–107 amino acids in length, were selected representing Gag (S1, S2, and S3), Pol (S4, S7, and S8), and Nef (S5 and S6) proteins from clade B (Table 1). The final linear TMEP-B sequence had a total length of 618 amino acids (~68 KDa) and the segments were ordered according to the full-length proteins found in the GPN fusion protein expressed by the MVA-B vaccine [20] (Figure 1A). At the N-terminus, we added a mutant form of the signal sequence for the human tissue plasminogen activator (tPA-22P/A SP), which has been shown to significantly improve the secretion of heterologous proteins [24] and a FLAG tag at the C-terminus for expression analysis. Single, dual, or triple alanine residues (A) were included as linker between segments in order to optimize the site of fission, to facilitate the processing of the fusion protein, and to decrease altered bioactivity of the protein moieties by the juxtaposition of the epitopes [11,12].

The TMEP-B immunogen contained 16 out of the 26 consensus clade B beneficial overlapping peptides defined by Mothe et al. that were predominantly targeted by subjects with superior HIV-1 control with exclusion of the non-beneficial peptides [25]. The missing beneficial epitopes were in p15, integrase, and Vif proteins that were not included in our immunogen. Similarly, it contained 9 out of the 13 conserved or cross-reactive epitopes preferentially targeted by the CD8^+^ T cells from Japanese individuals who controlled HIV-1 infection [26]. The S2 and S3 segments of TMEP-B covered six out of the seven conserved elements (CE) identified in p24-Gag by Rolland et al., which together cover >99% of the HIV-1 Group M sequences [27]. Cellular responses to some of these CE were significantly higher among patients able to control HIV-1 infection [28]. In terms of number of relevant human CD8^+^ and CD4^+^ T cell epitopes, the TMEP-B construct contained 90 out of the 181 CTL/CD8^+^ epitopes included in the “A list” and 174 out of the 348 T helper CD4^+^ epitopes defined for Gag, Pol, and Nef HIV-1 proteins [29]. Segments S1, S2, and S3 covered 56 out of the 72 (77.8%) CTLs and 146 out of the 230 (63.5%) T helper CD4^+^ epitopes described for Gag antigen; segments S4, S7, and S8 covered 24 out of the 68 (35.3%) CTLs and 22 out of the 78 (28.2%) T helper CD4^+^ epitopes described for Pol antigen; and S5 and S6 covered 10 out of the 41 (24.4%) CTLs and 6 out of the 40 (15%) T helper CD4^+^ epitopes described for Nef antigen. Among the T helper CD4^+^ epitopes, TMEP-B construct contained five out of the eight immunodominant peptides described by Kaufmamm et al., which were recognized at least one time by 93% of the seropositive subjects who had HIV-1-specific CD4 T cell responses. The most frequently targeted peptide, p24-Gag YVDRFYKTLRAEQASQEV, recognized by 58% of the study participants [30], showed the highest level of promiscuity with 14 distinct HLA-DR restrictions [29] and has been recently associated with HIV control in the context of HLA class II DBR*1101 [31]. Furthermore, the included immunodominant CD4^+^ epitopes were also targeted at a high frequency in acute HIV infection and the Gag-specific CD4 T cell responses in this phase inversely correlated in a significant way with viral set point in chronic HIV infection [32].

Besides the conserved regions, we also considered the inclusion of those epitope-dense regions or “hot spots” described for Gag, Pol, and Nef proteins that can bind to maximum number of HLA molecules in order to cover the global population HLA diversity [33]. The final TMEP-B sequence included CTL/CD8^+^ epitopes restricted by at least 64 different HLA class I alleles, as well as T helper/CD4^+^ epitopes restricted by about 30 different HLA class II presenting molecules.

Additionally, we used the sequences available for all variants of HIV-1 Gag, Pol, and Nef proteins to screen for the presence of all CTL/CD8^+^ and T helper/CD4^+^ epitopes reported to date. The search results were classified according to the characteristics of the source of patients as belonging to the general HIV-1-infected population, LTNP patients, or to our protein construct TMEP-B. This enabled us to compute the relative frequencies of appearance of each epitope in these populations and to identify epitope sequences that are more or less represented in LTNP patients than in the general HIV-1-infected cohort. Sixty-two epitopes were found to be under-represented in LTNP patients (their relative frequency was lower than in the general HIV-1-infected population), while 557 epitopes were over-represented in LTNP patients. TMEP-B sequence contains matches to 167 epitopes, of which 166 correspond to epitopes that are 5 to 25 times more frequent in LTNP patients (Tables S1 and S2).

### 3.2. Expression of TMEP-B Protein in Human Cells

The correct expression of FLAG-tagged TMEP-B protein from the recombinant DNA vector (DNA-TMEP) was evaluated from cell extracts and supernatants of infected and/or transiently transfected 293T cells by Western blot probed with an anti-FLAG antibody (Figure 1C). As the DNA vector contains a T7 promoter, for virus infection we used a vaccinia virus (VACV) vector from the WR strain expressing T7 polymerase (VT7). Similar levels of TMEP-B protein were detected at the expected size (~68 KDa) in extracts from cells either infected with the wild type VACV WR virus and transfected with DNA-TMEP (lane 1) or only transfected with DNA-TMEP vector driven by the CMV promoter (lane 3). However, in the extract from cells infected with the recombinant virus VT7 and transfected with DNA-TMEP (lane 2), where the T7 RNA polymerase expressed by the recombinant virus is able to bind to the T7 promoter located upstream of the TMEP-B gene in the plasmid pcDNA-TMEP-B, a major increase in the expression level of TMEP-B protein was observed. In this extract, additional bands were also observed possibly reflecting products processed by post-translational modifications. Although we added the mutant form of the signal sequence for the human tissue plasminogen activator (tPA-22P/A SP) at the N-terminus of TMEP-B to improve secretion of the protein, we were unable to detect the TMEP-B product in the extracellular compartment (data not shown).

### 3.3. Cellular Immune Profile Induced by the DNA-TMEP Immunogen

#### 3.3.1. Magnitude, Breadth, and Functional Profile of the T Cell Responses Induced by DNA-TMEP in Homologous or Heterologous Prime/Boost Immunization Regimen with MVA-B

Once we verified the correct expression of TMEP-B protein in vitro, we decided to characterize in vivo the T cell immune responses induced by the DNA-TMEP vector following homologous or heterologous combination with the recombinant MVA-B vaccine, which simultaneously expresses the monomeric gp120 and the artificial budding defective 1326 aa read-through GPN fusion protein, both from clade B. For comparison purposes, we included a DNA-GPN vector expressing the same full-length GPN fusion protein expressed by MVA-B.

**Figure 2:**
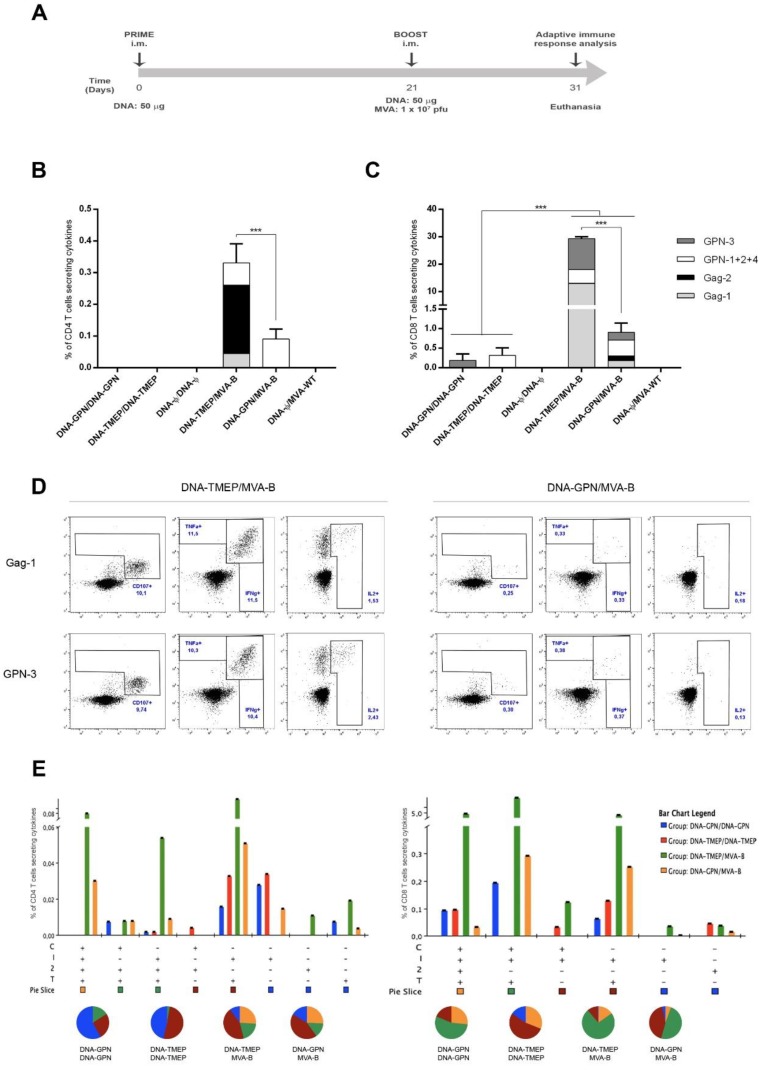
Polyfunctional Gag–Pol–Nef (GPN)-specific CD4 and CD8 T cell immune responses elicited in spleen after prime/boost immunization of mice with TMEP-B construct and modified vaccinia virus Ankara (MVA)-B recombinant virus. (**A**) Immunization schedule. Groups of 6–8-week-old female mice (*n* = 4) received 50 μg of pcDNA-_IIIB_GPN, pcDNA-TMEP-B, or pcDNA-φ by bilateral intramuscular route (i.m.); three weeks later, they received a bilateral i.m. inoculation of 50 μg of DNA (pcDNA-_IIIB_GPN, pcDNA-TMEP-B, or pcDNA-φ) or 1 × 10^7^ pfu of MVA-wild type (WT) or MVA-B viruses. At 10 days after the last immunization, mice were sacrificed and spleens were processed for intracellular cytokine staining (ICS) assay to analyze Gag–Pol–Nef-specific T cell immune responses. Two independent experiments have been performed for the different groups. Magnitude of the Gag- and GPN-specific CD4 (**B**) or CD8 (**C**) T cell immune responses were measured at 10 days post-boost by ICS assay after stimulation of splenocytes from immunized animals with the different HIV-1 clade B peptide pools. The total value in each group indicates the sum of the percentages of CD4^+^ or CD8^+^ T cells secreting CD107a and/or IFN-γ and/or IL-2 and/or TNF-α against Gag/GPN peptide pools. Data are background subtracted. *** *p* < 0.001. (**D**) Flow cytometry profiles of vaccine-induced CD8 T cell responses against Gag-1 or GPN-3 peptide pools in the groups immunized with the heterologous combinations DNA/MVA. (**E**) Functional profile of the Gag/GPN-specific CD4 (left panel) or CD8 (right panel) T cell responses in the different immunization groups. Positive combinations of the responses are represented on the *x* axis, whereas the percentages of the functionally distinct cell populations within the total CD4 or CD8 T cells are shown on the *y* axis. Responses are grouped and colour-coded based on the number of functions. Non-specific responses obtained in the control groups were subtracted in all populations. C: CD107a; I: IFN-γ; 2: IL-2; T: TNF-α.

BALB/c mice, four in each group, were immunized as described in Materials and Methods (immunization schedule depicted in Figure 2A) and adaptive T cell immune responses were analyzed by polychromatic ICS assay 10 days after the last immunization. To detect the HIV-1-specific T cell responses, splenocytes from immunized animals were stimulated ex vivo for 6 h with a panel of 450 peptides (15-mers overlapping by 11 amino acids) from HIV-1 clade B consensus, spanning the full-length sequences of Env, Gag, Pol, and Nef proteins included in the MVA-B vector. The peptides were grouped in seven pools as previously described: Env, Gag-1, Gag-2, GPN-1, GPN-2, GPN-3, and GPN-4; however, we decided to combine GPN-1, GPN-2, and GPN-4 pools as a unique stimulus (GPN-1+2+4) based on results from previous in vivo assays where the four GPN peptide pools were assayed individually (data not shown). Therefore, the HIV-1-specific CD4 and CD8 T cell responses were detected using the Env, Gag-1, Gag-2, GPN-1+2+4, and GPN-3 pools. Vector-specific responses were detected using the VACV E3_140-148_ peptide. The percentages of T cells with CD4 or CD8 phenotype that produced IFN-γ and/or IL-2 and/or TNF-α and/or CD107a established the overall CD4^+^ or CD8^+^ T cell immune responses. Mice primed with sham DNA (DNA-φ) and boosted with either DNA-φ or non-recombinant MVA-WT were used as controls.

First, we analyzed the HIV-1-specific T cell immune responses elicited in the different immunization groups. The overall magnitude of the HIV-1-specific CD4 and CD8 T cell responses was determined as the sum of the individual responses obtained for Gag-1, Gag-2, GPN-1+2+4, and GPN-3 peptide pools. As shown in Figure 2B,C, the vaccine-elicited immune response using homologous DNA/DNA or heterologous DNA/MVA prime/boost approach was mediated largely by the CD8 T cell subset. No HIV-1-specific CD4 T cell response was detected after homologous immunization and the overall HIV-1-specific CD4 T cell response elicited by the heterologous DNA-TMEP/MVA-B and DNA-GPN/MVA-B immunizations were 88- and 10-fold lower than their CD8 T cell response counterparts, respectively (Figure 2B,C). However, between them, the DNA-TMEP prime induced higher magnitude and broader HIV-1-specific CD4 T cell response than DNA-GPN when combined with MVA-B virus (Figure 2B). CD4 T cells from animals primed with DNA-TMEP recognized three out of the four HIV-1 peptide pools assayed and the individual contribution of each pool to the overall CD4 response was Gag-2 (65.2%) > GPN-1+2+4 (21.3%) > Gag-1 (13.5%), whereas in the group primed with DNA-GPN, the CD4 T cells only recognized the GPN-1+2+4 peptide pool.

With regard to the HIV-1-specific CD8 T cell responses, we observed that the heterologous combinations induced significantly higher magnitude and broader specific responses compared with the homologous regimen (*p* < 0.001) (Figure 2C). In the group of mice immunized with DNA-TMEP/MVA-B, the magnitude of the HIV-1-specific CD8 T cell response was 30-fold higher than that obtained in the group of animals receiving DNA-GPN/MVA-B (*p* < 0.001), indicating a potent effect of the optimized TMEP-B construct as a prime in the outcome of the T cell immune response compared with the full-length GPN protein. The CD8 T cells from animals receiving both heterologous regimens recognized all the HIV-1 pools assayed; however, their individual contribution to the overall specific response was different. In DNA-TMEP-primed mice boosted with MVA-B, the ranking was Gag-1 (43.8%) > GPN-3 (38.5%) > GPN-1+2+4 (17.2%) > Gag-2 (0.5%), whereas in DNA-GPN-primed mice boosted with MVA-B, it was GPN-1+2+4 (44.7%) > GPN-3 (21.7%) > Gag-1 (20.3%) > Gag-2 (13.3%), reflecting the different processing and presentation processes that both proteins undergo. Representative flow cytometry profiles of vaccine-induced CD8 T cell responses against Gag-1 or GPN-3 peptide pools in the groups immunized with the heterologous DNA prime/MVA boost regimens are shown in Figure 2D.

The quality of a T cell response can be characterized by the profile of cytokine production and cytotoxic potential. Based on the analysis of IFN-γ, IL-2, and TNF-α secretion and surface mobilization of CD107a on activated T cells as an indirect marker of cytotoxicity, eight different positive HIV-1-specific CD4 T cell populations (Figure 2E, left panel) and six different positive HIV-1-specific CD8 T cell populations (Figure 2E, right panel) were induced after immunization with the different vector combinations. The polyfunctional profile of the HIV-1-specific CD4 T cell responses in the groups immunized with the heterologous DNA/MVA combinations was similar, with 45% (DNA-TMEP/MVA-B) or 35% (DNA-GPN/MVA-B) of CD4^+^ T cells exhibiting three or four functions (Figure 2E, left panel). CD4 T cells producing CD107a+IFN-γ+IL-2+TNF-α, IFN-γ+IL-2+TNF-α, or IFN-γ+TNF-α were the most representative populations induced. HIV-1-specific CD8 T cell responses were more polyfunctional in the groups immunized with the heterologous DNA/MVA combinations than in the group immunized with the homologous DNA-TMEP/DNA-TMEP combination (data not shown), with 85% (DNA-TMEP/MVA-B) or 55% (DNA-GPN/MVA-B) of CD8^+^ T cells exhibiting three or four functions (Figure 2E, right panel). CD8 T cells producing CD107a+IFN-γ+IL-2+TNF-α, CD107a+IFN-γ+TNF-α, or IFN-γ+TNF-α were the most representative populations elicited by heterologous DNA/MVA immunization.

#### 3.3.2. Characterization of HIV-1-Specific Tfh Cell Response Induced by DNA-TMEP in Homologous or Heterologous Prime/Boost Immunization Regimen

Follicular helper T cells (Tfh) are critical for the generation of high-affinity Germinal Center (GC) B cells and for the interzonal back and forth migration of B cells for repeated rounds of somatic hypermutation (SHM). Functionally, Tfh cells help B cells by delivering signals via costimulatory molecules and cytokines (CD40L, IL-21, IL-4, and CXCL13) that constitute the functional signature of this specific CD4 T cell subset [34].

**Figure 3:**
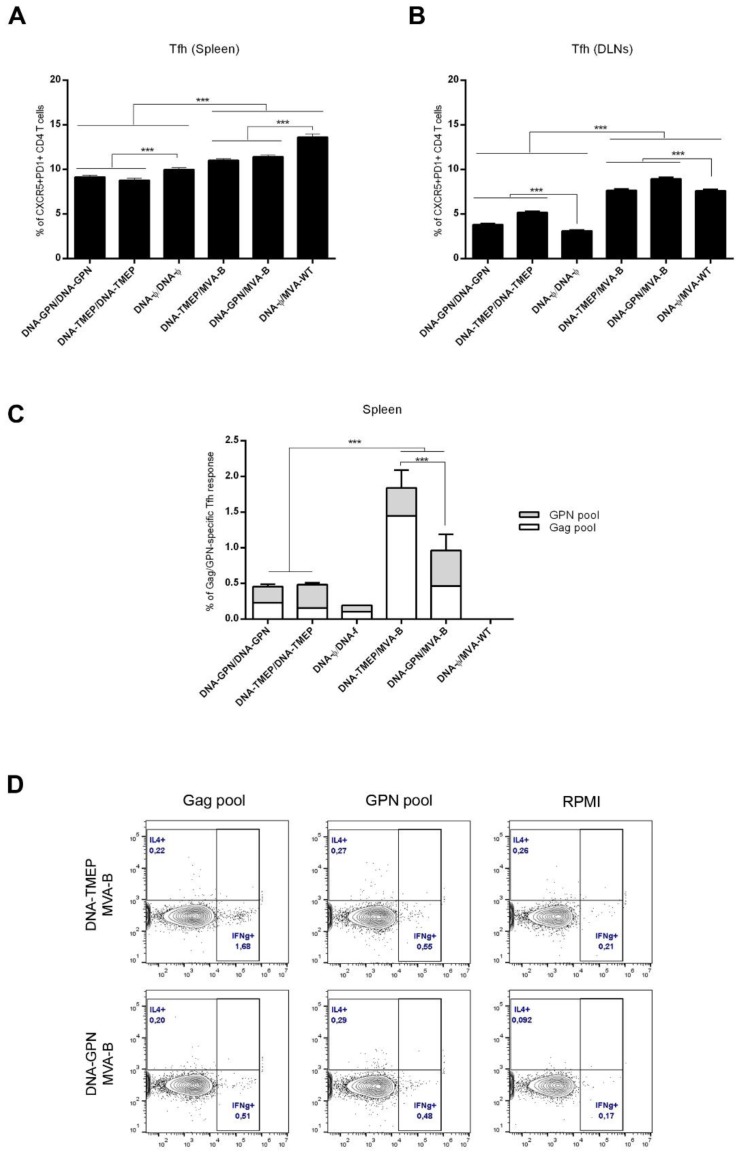
Gag–Pol–Nef-specific Tfh T cell immune responses elicited in spleen and draining lymph nodes (DLNs) after prime/boost immunization of mice with TMEP-B construct and MVA-B virus. Magnitude of the total CD4 T cells with Tfh phenotype (CXCR5^+^PD1^+^) in spleen (**A**) and DLNs (**B**) measured 10 days after the last immunization by ICS assay in the non-stimulated samples (RPMI). *** *p* < 0.001. (**C**) Magnitude of the Gag/GPN-specific Tfh cells in spleen measured 10 days after the last immunization by ICS assay following stimulation of splenocytes derived from immunized animals with the different HIV-1 clade B Gag and GPN peptide pools. The total value in each group indicates the sum of the percentages of Tfh^+^ T cells secreting IL-4 and/or IFN-γ against Gag/GPN peptide pools. Data are background subtracted. *** *p* < 0.001. (**D**) Flow cytometry profiles of vaccine-induced Tfh cell responses in spleen against Gag and GPN peptide pools in the groups immunized with the heterologous combinations DNA/MVA.

Because frequency and quality of Tfh cells have been previously correlated with the development of broadly neutralizing antibodies (bNAbs) [35,36], we decided to characterize this specific cellular subset in the spleen and DLNs of immunized mice. Splenocytes or DLN lymphocytes from immunized animals were non-stimulated (RPMI) or stimulated ex vivo for 6 h with the Env, Gag (Gag-1+Gag-2), and GPN (GPN-1+GPN-2+GPN-3+GPN-4) HIV-1 peptide pools. The total number of Tfh CD4 T cells was determined in the non-stimulated samples by the expression of CXCR5 and PD1 (CXCR5^+^PD1^+^) surface markers. In spleen, the frequency of total Tfh CD4 T cells was higher than in DLNs. Furthermore, in both organs, the frequencies of total Tfh cells were dependent on the immunization protocol used, as higher values were detected in animals immunized with the heterologous DNA/MVA combinations compared with those observed in animals receiving the homologous DNA/DNA regimen (*p* < 0.001) (Figure 3A,B).

Interestingly, more than 70% of Tfh CD4 T cells were positive for both IL-21 and CD40L (CD154), independent of the stimulation used (data not shown). For this reason, the HIV-1-specific Tfh response was established by the percentages of IFN-γ and/or IL-4 producing CD4^+^CXCR5^+^PD1^+^ Tfh-like cells after HIV-1 peptide pool stimulation in comparison with non-antigen stimulation (RPMI). We only detected measurable HIV-1-specific Tfh response in spleen (Figure 3C). In this organ, the magnitude of the responses in the groups immunized with the heterologous combination was significantly higher than that obtained in the groups immunized with the homologous combination (*p* < 0.001). The overall magnitude of the HIV-1-specific Tfh cell response was significantly higher in the group of animals receiving DNA-TMEP/MVA-B immunization compared with that obtained in the group immunized with DNA-GPN/MVA-B (*p* < 0.001). Moreover, in DNA-TMEP-primed mice, the HIV-1-specific Tfh response was mainly directed against Gag pool [Gag (69.3%) > GPN (30.4%)] because in DNA-GPN-primed mice, the specific response was more balanced, but mainly directed against GPN pool [GPN (56.9%) > Gag (43.1%)]. Representative flow cytometry profiles of vaccine-induced Tfh cell responses against Gag or GPN peptide pools in the groups immunized with the heterologous DNA prime/MVA boost regimens are shown in Figure 3D.

#### 3.3.3. Effect of DNA Prime on the Env-Specific T Cell Immune Responses in Animals Receiving MVA-B Boost

**Figure 4:**
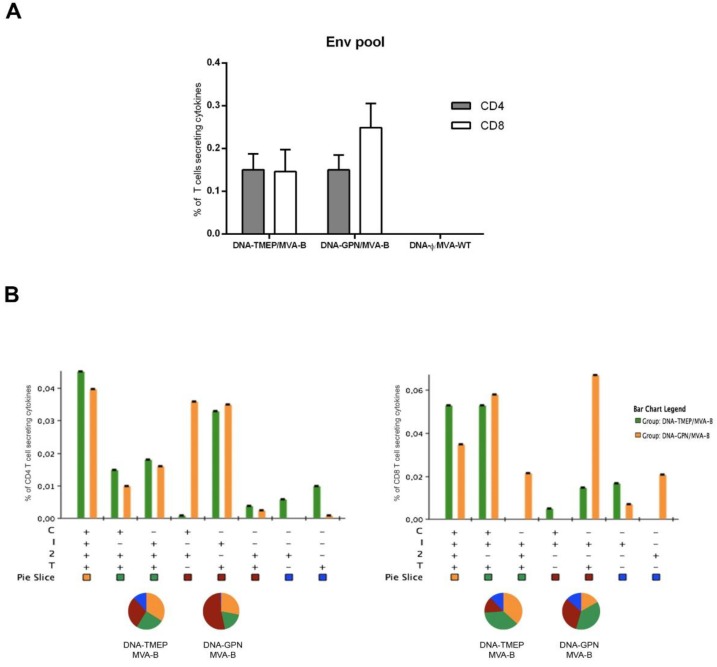

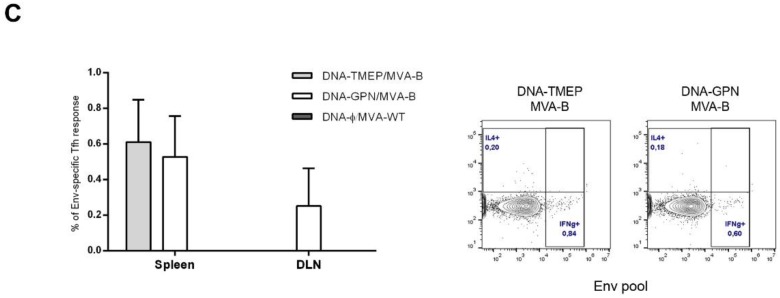
Env-specific T cell immune responses (CD4, CD8, and Tfh) elicited in spleen and DLNs after prime/boost immunization of mice with TMEP-B construct and MVA-B virus. (**A**) Magnitude of the Env-specific CD4 or CD8 T cell immune responses measured at 10 days post-boost by ICS assay after stimulation of splenocytes from immunized animals with HIV-1 clade B Env peptide pool. The total value in each group indicates the sum of the percentages of CD4^+^ or CD8^+^ T cells secreting CD107a and/or IFN-γ and/or IL-2 and/or TNF-α against Env peptide pool. Data are background subtracted. (**B**) Functional profile of the Env-specific CD4 (left panel) or CD8 (right panel) T cell responses in the different immunization groups. Positive combinations of the responses are represented on the *x* axis, whereas the percentages of the functionally distinct cell populations within the total CD4 or CD8 T cells are shown on the *y* axis. Responses are grouped and colour-coded based on the number of functions. Non-specific responses obtained in the control groups were subtracted in all populations. C: CD107a; I: IFN-γ; 2: IL-2; T: TNF-α. (**C**, left panel) Magnitude of the Env-specific Tfh cells in spleen and DLNs measured as in (**A**). The total value in each group indicates the sum of the percentages of Tfh^+^ T cells secreting IL-4 and/or IFN-γ against Env pool. Data are background subtracted. (**C**, right panel) Flow cytometry profiles of vaccine-induced Tfh cell responses in spleen against Env pool in the groups immunized with the heterologous combinations DNA/MVA.

Finally, we decided to evaluate to what extent the T cell activation induced by the DNA vectors expressing either TMEP-B or the full-length GPN proteins might impact on the magnitude and quality of the T cell immune response elicited against the Env antigen, only expressed when MVA-B was administered in the boost. As shown in Figure 4A, the Env-specific T cell response was equally distributed between CD4 and CD8 compartments in animals primed either with DNA-TMEP or DNA-GPN, although in DNA-GPN-primed animals, there was a trend towards higher CD8 T cell contribution (not statistically significant).

When we evaluated the polyfunctional profile of the Env-specific response, we observed that eight and seven different positive CD4 (Figure 4B, left panel) and CD8 (Figure 4B, right panel) T cell populations, respectively, were induced after immunization with the different DNA/MVA combinations. Env-specific CD4 T cell responses were more polyfunctional in animals primed with DNA-TMEP compared with animals primed with DNA-GPN, with 60% (DNA-TMEP/MVA-B) or 45% (DNA-GPN/MVA-B) of CD4^+^ T cells exhibiting three or four functions (Figure 4B, left panel). CD4 T cells producing CD107a+IFN-γ+IL-2+TNF-α or IFN-γ+TNF-α were the most representative populations induced. Similarly, Env-specific CD8 T cell responses were also more polyfunctional in animals primed with DNA-TMEP compared with animals primed with DNA-GPN, with 75% (DNA-TMEP/MVA-B) or 55% (DNA-GPN/MVA-B) of CD8^+^ T cells exhibiting three or four functions (Figure 4B, right panel), respectively. CD8 T cells producing CD107a+IFN-γ+IL-2+TNF-α or CD107a+IFN-γ+TNF-α were the most representative populations induced in both DNA/MVA groups, with the additional population IFN-γ+TNF-α highly induced in DNA-GPN/MVA-B immunization group.

Regarding the Env-specific *Tfh* CD4 T cell response induced in the secondary lymphoid tissues (spleen and DLNs) of immunized mice, we observed that both DNA vectors induced similar Tfh response in the spleen; however, in DLNs, we were able to detect positive response only in animals primed with DNA-GPN (Figure 4C, left panel). Representative flow cytometry profiles of vaccine-induced Tfh CD4 T cell responses against Env pool in the groups immunized with the heterologous combinations DNA/MVA are shown in Figure 4C, right panel.

Altogether these data confirmed that heterologous DNA prime/MVA boost protocol was more efficient in activating HIV-1-specific T cell immune responses than the homologous DNA/DNA combination. Moreover, DNA-TMEP immunogen administered as a prime when combined with MVA-B as a boost was able to induce robust, broad, and polyfunctional HIV-1-specific T cell immune responses, mainly mediated by CD8 T cells and primarily directed against Gag antigen.

#### 3.3.4. T Cell Immune Response against VACV E3 Peptide Induced by DNA/MVA Heterologous Combination

**Figure 5:**
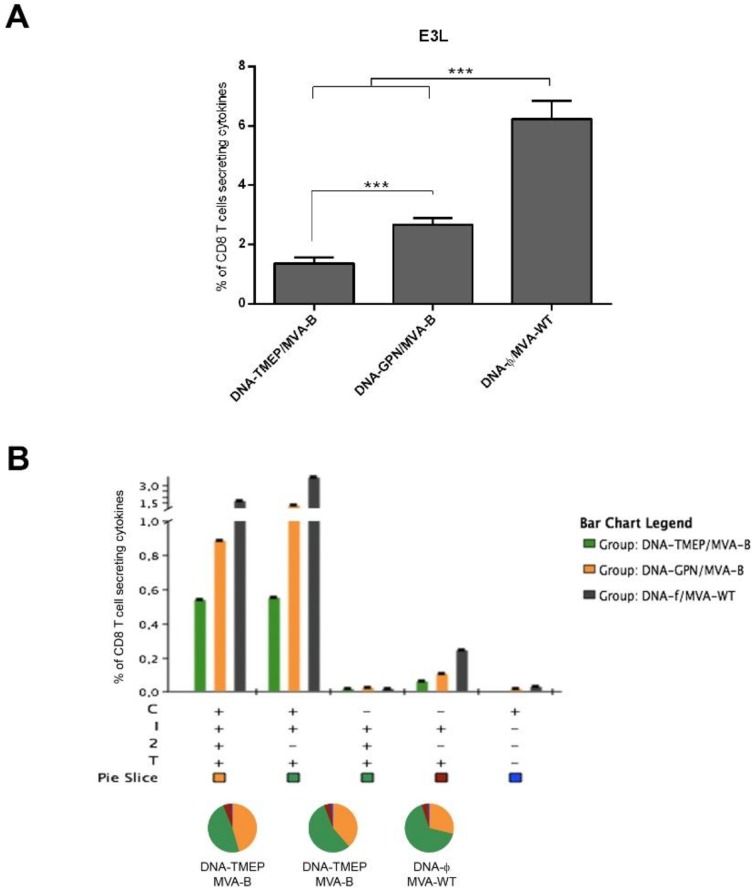

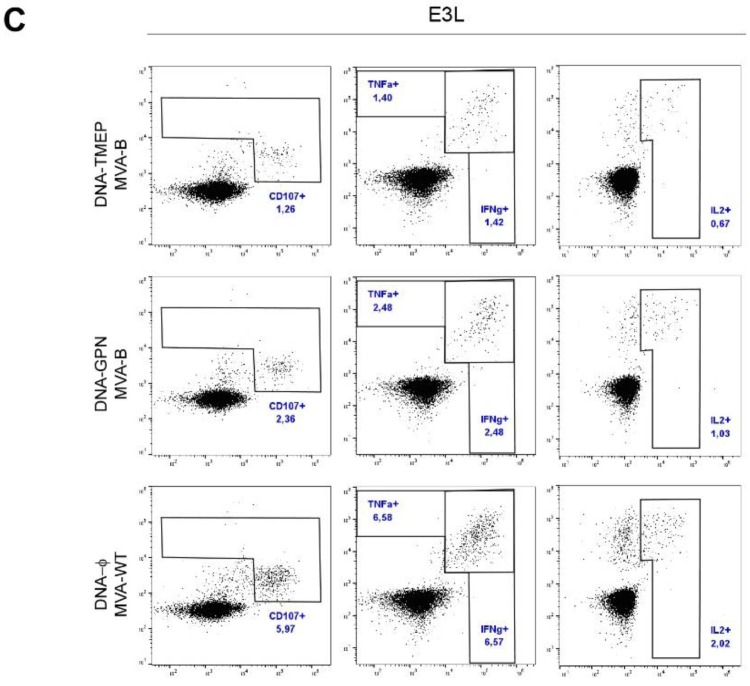
VACV vector E3-specific CD8 T cell immune responses elicited in spleen after prime/boost immunization of mice with TMEP-B construct and MVA-B virus. (**A**) Magnitude of the E3-specific CD8 T cell immune responses measured at 10 days post-boost by ICS assay after stimulation of splenocytes from immunized animals with VACV E3 peptide. The magnitude in each group indicates the sum of the percentages of CD8^+^ T cells secreting CD107a and/or IFN-γ and/or IL-2 and/or TNF-α against E3 peptide. Data are background subtracted. *** *p* < 0.001. (**B**) Functional profile of the E3-specific CD8 T cell responses in the different immunization groups. Positive combinations of the responses are represented on the *x* axis, whereas the percentages of the functionally distinct cell populations within the total CD8 T cells are shown on the *y* axis. Responses are grouped and colour-coded based on the number of functions. C: CD107a; I: IFN-γ; 2: IL-2; T: TNF-α. (**C**) Flow cytometry profiles of vaccine-induced CD8 T cell responses in spleen against E3 peptide in the groups immunized with the heterologous combinations DNA/MVA.

In the same way, we also evaluated the effect of the DNA prime on the magnitude and quality of the anti-VACV T cell immune response elicited when MVA-B vaccine was administered to the animals in the boost. We observed that the magnitude of the E3-specific CD8 T cell responses was lower in the groups of animals primed with DNA-TMEP or DNA-GPN compared with the control DNA-φ/MVA-WT immunization group (*p* < 0.001) (Figure 5A). Furthermore, a significant reduction of the anti-vector CD8 T cell response was detected in animals primed with DNA-TMEP compared with DNA-GPN-primed animals (*p* < 0.001) (Figure 5A), suggesting an inverse correlation between HIV-1- and VACV-specific T cell immune responses. In spite of the differences in the magnitude of the response between the groups, the polyfunctional profile was similar, with 90% of CD8^+^ T cells exhibiting three or four functions (Figure 5B). CD8 T cells producing CD107a+IFN-γ+IL-2+TNF-α or CD107a+IFN-γ+TNF-α were the most representative populations induced by the heterologous combination. Representative flow cytometry profiles of vaccine-induced CD8 T cell responses against VACV E3 peptide in the groups immunized with the heterologous combinations DNA/MVA are shown in Figure 5C.

### 3.4. Humoral Immune Profile against HIV-1 Gag-P24

**Figure 6:**
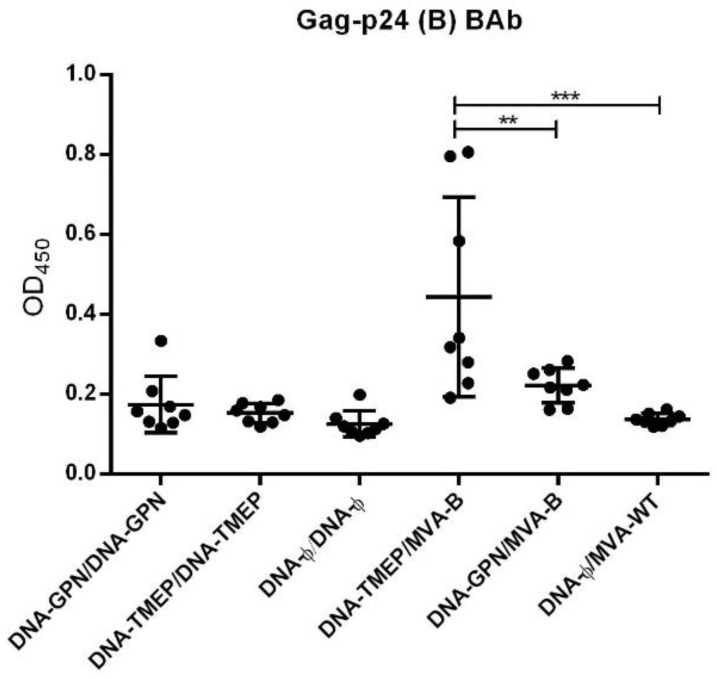
Anti-p24 humoral responses elicited in serum from immunized mice after prime/boost immunization with TMEP-B construct and MVA-B virus. The graph represents the values of optical density at 450 nm (OD_450_) for each animal at a serum dilution of 1:50 (dots) and the mean value (solid line) and standard deviation of each group. ** *p* < 0.005; *** *p* < 0.001.

While TMEP-B construct was designed as a T cell immunogen and the full-length GPN fusion protein was expressed by either DNA-GPN or MVA-B as an intracellular antigen, we decided to evaluate the effect of both constructions (TMEP-B and GPN) in eliciting Gag-p24-specific humoral immune responses. We quantified by ELISA the Gag-p24-specific IgG levels in individual sera from mice immunized with the different homologous and heterologous vector combinations in two independent in vivo assays. As shown in Figure 6, the levels of anti-Gag-p24 detected were low, but the highest response was obtained in the group immunized with DNA-TMEP/MVA-B followed by the group immunized with the heterologous combination DNA-GPN/MVA-B.

## 4. Discussion

The development of an effective HIV vaccine largely depends on our understanding of the complex dynamics that exist between the host immune response and viral adaptation to selective pressure exerted by the host. It has been reported that as in natural infection, vaccines encoding full- or near full-length HIV-1 proteins when administered to HIV-1-naïve or primed individuals [37], and also to non-human primates [9,38], elicited responses that are biased towards non-beneficial targets. Vaccine-induced immune responses preferentially target variable non-protective epitopes masking the immune responses to the protective conserved ones. Recently, different approaches are being implemented in the vaccine development field to overcome viral diversity and natural immunodominance hierarchies. Among them, the rational design of T cell multiepitopic immunogens, including the most conserved T cell regions within the viral proteome that are associated with HIV control, have revealed promising results in pre-clinical and clinical settings [11,12,14,15].

In this study, we applied the relevant findings obtained using T cell immunogens in the design of the TMEP-B construct. As our main objective was the generation of an alternative candidate to be combined with the MVA-B vaccine to maximize the vaccine-induced immunity and overcome the immunodominance of Env responses detected in clinical studies, the HIV-1 antigens targeted for the TMEP-B design were Gag, Pol, and Nef. We selected regions within each of these proteins that were both conserved across all clades and enriched for beneficial CD8^+^ and CD4^+^ T cell epitopes, restricted by a wide range of HLA class I and II molecules, which have been functionally associated with low viral load and HIV control. TMEP-B protein was vectored by DNA (DNA-TMEP) and evaluated in mice when administered in homologous or heterologous prime/boost regimens in combination with MVA-B vaccine. Although regions included in the TMEP-B construct were selected based upon human epitope data, some of the Gag and Pol CTL and CD4 T cell epitopes included in the final construction have been previously described and mapped as H-2^d^-restricted immunogenic peptides recognized by the BALB/c mice, thus providing additional tools for studying and optimizing vaccine regimens in this commonly used small animal model [39].

We observed that heterologous DNA-TMEP prime followed by MVA-B boost regimen induced higher magnitude of HIV-1-specific CD8 T cells with broader epitope recognition and higher polyfunctional profile than the homologous combination DNA-TMEP/DNA-TMEP or the heterologous regimen DNA-GPN/MVA-B, in which both vectors shared the expression of the same full-length GPN fusion protein. These results suggest that the use of optimized TMEP-B immunogen as a prime vector preferentially focused the immune responses to T cell clones targeting conserved protective epitopes that were difficult to prime when full-length antigens were used because of the immunodominance hierarchy. The DNA-TMEP-primed responses were efficiently expanded after MVA-B boost expressing not only the related full-length GPN antigen, but also the Env monomeric gp120. Similar results were reported in non-human primates using DNA-based immunogens encoding highly conserved sequences within HIV-1 Gag-p24 (CE DNA). It was observed that priming with CE DNA followed by booster with CE+full-length p55*gag* DNA was the optimal regimen that maximized the magnitude and breadth of the CE immunity [9,38]. Furthermore, it has been recently reported that the T cell responses induced by the CE DNA vaccine were long-lasting (~2 years) and effectively recalled upon a single booster vaccination using either CE DNA or a recombinant MVA expressing the full-length Gag/Pol + Env HIV-1 antigens [40]. In the last case, the expression of additional immunogens by MVA had no negative impact on boosting the responses targeting the conserved elements indicating that priming with immunogens based on conserved regions permanently altered the immune hierarchy.

In addition, DNA-TMEP prime induced higher magnitude and broader HIV-1-specific CD4 T cell response than DNA-GPN when combined with MVA-B vaccine, which is in accordance with the inclusion of highly immunogenic CD4 T cell epitopes in the TMEP-B construct. Both HIV-1-specific CD4 and CD8 T cell responses obtained in animals immunized with DNA-TMEP/MVA-B were mainly directed against Gag. Such results are considered as favorable hallmarks for HIV protection because the induction of Gag-specific responses during acute or chronic HIV infection has demonstrated to have an active role in the control of viremia [26,28,31,32,41]. The segments from HIV-1 Gag proteins included in the TMEP-B construct contained a short region within p24 targeted by HIV-1-specific CD8 T cell responses that were associated with better disease outcome [41]. Interestingly, this region, as well as proximal regions also included in our construct, was also preferentially targeted by HIV-1-specific CD4 T cell responses associated with slower disease progression [32], supporting the hypothesis that HIV-1-specific CD4 T cell responses may support these efficient HIV-1-specific CD8 T cell responses.

During the last few years, Tfh subset has been extensively studied in the settings of vaccination or chronic HIV infection. The partly efficacious RV144 HIV vaccine induced higher levels of HIV-1-specific circulating Tfh (cTfh) cells, defined by IL-21 production, than other HIV vaccine candidates that had shown no efficacy [42]. In HIV-1-infected patients was also reported an association between the proportion of PD-1^+^ cTfh or PD-1^+^CXCR3^−^ cTfh and the induction of broadly neutralizing antibodies [43]. Moreover, a high frequency of HIV-1-specific cTfh cells has been recently associated with preserved memory B cell responses in HIV controllers [44]. Altogether, all these findings suggested a key role for the Tfh response in inducing protective responses against HIV. Here, we observed that the overall magnitude of the HIV-1-specific Tfh cell response was significantly higher in the splenocytes from animals receiving DNA-TMEP/MVA-B immunization compared with the group immunized with DNA-GPN/MVA-B, revealing the potential of the TMEP-B prime to induce this relevant cell subset.

The analysis of the Env-specific T cell response (CD4, CD8, and Tfh T cells) after a single MVA-B boost showed that the magnitude of this response was similar in animals primed with DNA-TMEP or DNA-GPN, although there was a trend to a lower Env-specific CD8 T cell response in DNA-TMEP-primed animals. Moreover, anti-VACV E3 CD8 T cell response was significantly lower in DNA-TMEP-primed animals, suggesting an inverse correlation between HIV-1- and vector-specific T cell immune responses. It has been reported that a critical event in the initiation of the immune response to vaccination is the primary activation of antigen-specific T cells, which severely influences both the magnitude and the quality of the immune response elicited by vaccination. Polarization of the distinct effector T cell subsets is indeed regulated by the strength of antigenic stimulation, as well as by the cytokines present during priming [45]. The expression of the heterologous HIV-1 antigens in the prime impacted the local innate immune responses, costimulatory and cytokine signals, during the antigen presentation event inducing the activation of HIV-1-specific T cell clones capable of responding to recall immunization. The strength of the induced HIV-1-specific T cell responses during primary responses seems to limit the priming of naïve cells specific for other antigens not included in the first immunization. In our study, the TMEP-B construction induced the highest and more polyfunctional HIV-1-specific responses and, therefore, the lowest anti-vector immunity.

HIV multiepitopic DNA vaccines have been poorly immunogenic in humans when used alone; however, they have demonstrated to efficiently prime the specific-responses when combined with a heterologous boost vector. DNA prime/MVA boost regimen has been widely reported to elicit potent, durable, and protective responses in both non-human primates and clinical trials [40,46,47,48]. What is the advantage of the DNA-TMEP/MVA-B protocol? This protocol could be implemented as an improvement of the HIV vaccine MVA-B that showed limited benefit in HIV-1-infected individuals under antiretroviral (ART) therapy [5]. While in MVA-B-vaccinated individuals, there was a trend towards the control of viral rebound after ART removal, this effect did not prevent virus re-emergence with time, suggesting the need for further improvements. In the past few years, a major interest has emerged towards the development of therapeutic procedures aimed at eliminating HIV from its reservoirs using a combination of approaches [49]. The use of DNA-TMEP/MVA-B could in fact contribute to the enhancement of the specific CD8 T cell population in an infected and vaccinated person that, together with the administration of broadly neutralizing antibodies and activators of viral reservoirs, could help in the control and elimination of virus-infected cells.

Overall, our studies established a potent HIV-1 immunization protocol based on priming with a novel multiepitopic antigen TMEP-B followed by a booster with the HIV vaccine MVA-B. This vaccination leads to activation of HIV-1-specific T cell responses (CD4, CD8, and Tfh), with preference for high levels of CD8^+^ T cells, immunological markers that might benefit the control of HIV infection. These findings extend and support the rational design of T cell immunogens, including conserved viral regions associated with HIV control, as a powerful component to be included in heterologous prophylactic or therapeutic vaccine regimens.

## Figures and Tables

**Table 1:** Characteristics of the eight HIV-1 segments included in the T cell multiepitopic peptide (TMEP-B) sequence. GPN: Gag–Pol–Nef; Prt: protease; RT: reverse transcriptase.

Segments	Length (aa)	Position on HXB2	HIV-1 Protein	Coverage by HIV-1 Clade B Consensus Peptide Pools
S1	85	17–101	Gag-p17	Gag-1
S2	72	138–209	Gag-p24	Gag-1
S3	104	259–362	Gag-p24	Gag-2
S4	107	101–207	Pol-Prt	GPN-1
S5	34	81–114	Nef	GPN-2
S6	13	56–68	Nef	GPN-2
S7	99	399–497	Pol-RT	GPN-3
S8	55	328–382	Pol-RT	GPN-4

## Supplementary Materials

The following are available online at http://www.mdpi.com/1999-4915/10/8/424/s1. Table S1: Relative frequencies of appearance of T helper/CD4^+^ epitopes in the general HIV-1-infected population (max.pop) and in long term non-progressor (LTNP) patients, Table S2: Relative frequencies of appearance of CTL/CD8^+^ epitopes in the general HIV-1-infected population (max.pop) and in long term non-progressor (LTNP) patients.
